# Supplementary material for: Heat adaptation of phage T7 under an extended genetic code
Source: Virus Evol. 2021 Dec 1;7(2):veab100. doi: 10.1093/ve/veab100 (PMC8923235; doi:10.1093/ve/veab100)
Supplement: veab100_Supp [file veab100_supp.zip › Sequencing_Analysis.zip!/Sequencing_Analysis/S1/evidence/JC_40.html]

BRESEQ :: Evidence


| New junction evidence | | | | | | | | | | | |
| --- | --- | --- | --- | --- | --- | --- | --- | --- | --- | --- | --- |
|  | | seq id | position | reads (cov) | reads (cov) | score | skew | freq | annotation | gene | product |
| \* | ? | NC\_001604.1 | = 281 | 3834 (1.030) | 908 (0.230) +TAAAGACCAGACC | 18/508 | 36.9 | 18.1% | intergenic (–/–) | *–/–* | –/– |
|  | ? | NC\_001604.1 | 282 = | 4288 (1.020) |  |  |  |  | intergenic (–/–) | *–/–* | –/– |
| Rejected: Coverage evenness skew score above cutoff. | | | | | | | | | | | |
| Rejected: Frequency below/above cutoff threshold. | | | | | | | | | | | |

Only 100 of 912 total aligned reads displayed.  

`‑‑‑‑‑‑‑‑‑‑‑‑‑TCTCACAGTGTACGGACCTAAAGTT‑CCCCCATA‑GGGGGTACCTAAAGCCCAGCCAATCACCTAAAGTCAACCTTCGGTTGACCTTGAGGGTTCCCTAAGGGTTGGGGATGACCCTTGGGTTTGTCTTTGGGTGTTACCTTGAGTGTCTCTCTGTGTCCCTATCTGTTACAGTCTCCTAAAGTATCCTCCTAAAGTCACCTCCTAACGTCCATCCTAAAGCCAACACCTAAAGCCTACACCTAAAGACCCATCAAGTCAACGCCTATCTTAAAGTTTAAACA‑‑‑‑‑‑‑‑‑‑‑‑‑‑‑‑‑‑‑‑‑‑‑‑‑‑‑‑‑‑‑‑‑‑‑‑‑‑‑‑‑‑‑‑‑‑‑‑‑‑‑‑‑‑‑‑‑‑‑‑‑‑‑‑‑‑‑‑‑‑‑‑‑‑‑‑‑‑‑‑‑‑‑‑‑‑‑‑‑‑‑‑‑‑‑‑‑‑‑‑‑‑‑‑‑‑‑‑‑‑‑‑‑‑‑‑‑‑‑‑‑‑‑‑‑‑‑‑‑‑‑‑‑‑‑‑‑‑‑‑‑‑‑‑‑‑‑‑‑‑‑‑‑‑‑‑‑‑‑‑‑‑‑‑‑‑‑‑‑‑‑‑‑‑‑‑‑‑‑‑‑‑‑‑‑‑‑‑‑‑‑‑‑‑‑‑‑‑‑‑‑‑‑‑‑‑‑‑‑‑‑‑‑‑‑‑‑‑‑‑‑‑‑‑‑‑‑‑‑‑‑‑‑‑‑‑‑‑‑‑‑‑‑‑‑‑‑‑‑‑‑‑‑‑‑‑‑‑‑‑‑‑‑‑‑‑‑‑‑‑‑‑‑‑‑‑‑‑‑‑‑‑‑‑‑‑‑‑‑‑‑‑‑‑‑‑‑‑‑‑‑‑‑  >  NC_001604.1/1‑281`  
`‑‑‑‑‑‑‑‑‑‑‑‑‑‑‑‑‑‑‑‑‑‑‑‑‑‑‑‑‑‑‑‑‑‑‑‑‑‑‑‑‑‑‑‑‑‑‑‑‑‑‑‑‑‑‑‑‑‑‑‑‑‑‑‑‑‑‑‑‑‑‑‑‑‑‑‑‑‑‑‑‑‑‑‑‑‑‑‑‑‑‑‑‑‑‑‑‑‑‑‑‑‑‑‑‑‑‑‑‑‑‑‑‑‑‑‑‑‑‑‑‑‑‑‑‑‑‑‑‑‑‑‑‑‑‑‑‑‑‑‑‑‑‑‑‑‑‑‑‑‑‑‑‑‑‑‑‑‑‑‑‑‑‑‑‑‑‑‑‑‑‑‑‑‑‑‑‑‑‑‑‑‑‑‑‑‑‑‑‑‑‑‑‑‑‑‑‑‑‑‑‑‑‑‑‑‑‑‑‑‑‑‑‑‑‑‑‑‑‑‑‑‑‑‑‑‑‑‑‑‑‑‑‑‑‑‑‑‑‑‑‑‑‑‑‑‑‑‑‑‑‑‑‑‑‑‑‑‑‑‑‑‑‑‑‑‑‑‑‑‑‑‑‑‑‑‑‑‑‑‑‑‑‑‑‑‑‑‑‑‑‑‑‑‑‑‑‑‑‑‑‑‑‑‑‑‑‑‑‑TAAAGACCAGACCTAAAGACCAGACCTAAAGACACTACATAAAGACCAGACCTAAAGACGCCTTGTTGTTAGCCATAAAGTGATAACCTTTAATCATTGTCTTTATTAATACAACTCACTATAAGGAGAGACAACTTAAAGAGACTTAAAAGATTAATTTAAAATTTATCAAAAAGAGTATTGACTTAAAGTCTAACCTATAGGATACTTACAGCCATCGAGAGGGACACGGCGAATAGCCATCCCAATCGACACCGGGGTCAACCGGATAAGTAGACAGCCTGATAAGT  >  NC_001604.1/282‑571`  

                                                                                                                                                                                                                                                                                                        `|||||||||||||`                                                                                                                                                                                                                                                                                                    
`gggctcggagatgttgataagagacaGGACCTAAAGTT‑CCCCCATA‑GGGGGTACCTAAAGCCCAGCCAATCACCTAAAGTCAACCTTCGGTTGGACTTGAGGGTTCCCTAAGGGTTGGGGATGACCCTTGGGTTTGTCTTTGGGTGTTACCTTGAGTGTCTCTCTGTGTCCCTATCTGTTACAGTCTCCTAAAGTATCCTCCTAAAGACACCTCCTAACGTCCATCCTAAAGCCAACACCTAAAGCCTACACCTAAAGACCCATCAAGTCAACGCCTATCTTAAAGTTTAAACATAAAGAC                                                                                                                                                                                                                                                                                                          <  1:634042/275‑1`  
`gggctcggagatgtgtataagagacaGGACCTAAAGTT‑CCCCCATA‑GGGGGTACCTAAAGCCCAGCCAATCAACTAAAGTCAACCTTCGGTTGACCTTGAGGGTGCCCTAAGGGTTGGGGATGACCCTTGGGTTTGTCTTTGGGTGTTACCTTGAGTGTCTCTCTGTGTCCCTATCTGTTACAGTCTCCTAAAGTATCCTCCTAAAGTCACCTCCTAACGTCCATCCTAAAGCCAACACCTAAAGCCTACACCTAAAGACCCATCAAGTCAACGCCTATCTTAAAGTTTAAACATAAAGAC                                                                                                                                                                                                                                                                                                          <  1:662518/275‑1`  
`gagatgtgtaaaagagtcagGTGGACGGACCTAAAGTT‑CCCCCAAA‑GGGGGTACCTAAAGCCCAGCCAATCACCTAAAGTCAACCTTCGGTTGACCTTGAGGGTTCCCTAAGGTTTGGGGATGACCCTTGGGTTTGTCTTTGGGTGTTAACTTGAGTGTCTCTCTGTGGCCCTATCTGTTACAGTCTACTAAAGTATCCTCCTAAAGTCACCTCCTAACGTCCATCCTAAAGCCAACACCTAAAGCCTACACCTAAAGACCCATCAAGTCAACGCCTATCTTAAAGTTTAAACATAAAGAC                                                                                                                                                                                                                                                                                                          <  1:387583/281‑1`  
`cggagatttgtataagagacagGTACGGACCTAAAGTT‑CCCCCATA‑GGGGGTACCTAAAGCCCAGCCAATCACCTAAAGTCAACCTTCGGTTGACCTTGAGGGTTCCCTAAGGGTTGGGGATGACCCTTGGGTTTGTCTTTGGGTGTTACCTTGAGTGTCTCTCTGTGTCCCTATCTGTTACAGTCTCCTAAAGTATCCTCCTAAAGTCACCTCCTAACGTCCATCCTAAAGCCAACACCTAAAGCCTACACCTAAAGACCCATCAAGTCAACGCCTATCTTAAAGTTTAAACATAAAGAC                                                                                                                                                                                                                                                                                                          <  1:398873/279‑1`  
 `tcttagtgactaTCTCACAGTGTACGGACCTAAAGTT‑CCCCCATA‑GGGGGTACCTAAAGCCCAGCCAATCACCTAAAGTCAACCTTCGGTTGACCTTGAGGGTTCCCTAAGGGTTGGGGATGACCCTTGGGTTTGTCTTTGGGTGTTACCTTGAGTGTCTCTCTGTGTCCCTATCTGTTACAGTCTCCTAAAGTATCCTCCTAAAGTCACCTCCTAACGTCCATCCTAAAGCCAACACCTAAAGCCTACACCTAAAGACCCATCAAGTCAACGCCTATCTTAAAGTTTAAACATAAAGACC                                                                                                                                                                                                                                                                                                         <  1:153689/289‑1`  
      `tgtgtataagagacagGTACGGACCTAAAGGT‑CCCCCATA‑GGGGGTACCTAAAGCCCAGCCAATCACCTAAAGTCAACCTTCGGTTGACCTTGAGGGTTCCCTAAGGGTTGGGGATGACCCTTGGGTTTGTCTTTGGGTGTTACCTTGAGTGTCTCTCTGTGTTCCTATCTGTTACAGTCTCCTAAAGTATCCTCCTAAAGTCACCTCCTAACGTCCATCCTAAAGCCAACACCTAAAGCCTACACCTAAAGACCCATCAAGTCAACGCCTATCTTAAAGTTTAAACATAAAGACCAGACC                                                                                                                                                                                                                                                                                                    <  1:282444/285‑1`  
      `tgtataagagacagGTGTACGGACCTAAAGTT‑CCCCCATA‑GGGGGTACCTAAAGCCCAGCCAATCACCTAAAGTCAACCTTCGGTTGACCTTGAGGGTTCCCTAAGGGTTGGGGATGACCCTTGGGTTTGTCTTTGGGTGTTACCTTGAGTGTCTCTCTGTGTCCCTATCTGTTACAGTCTCCTAAAGTATCCTCCTAAAGTCACCTCCTAACGTCCATCCTAAAGCCAACACCTAAAGCCTACACCTAAAGACCCATCAAGTCAACGCCTATCTTAAAGTTTAAACATAAAGACCAGACC                                                                                                                                                                                                                                                                                                    <  1:9339/287‑1`  
      `tgtataagagaaagGTGTACGGACCTAAGGTT‑CCCCCATA‑GGGGGTACCTAAAGCCCAGCCAATCACCTAAAGTCAACCTTCGGTTGACCTTGAGGGTTCCCTAAGGGTTGGGGATGACCCTTGGGTTTGTCTTTGGGTGTTACCTTGAGTGTCTCTCTGTGTCCCTATCTGTTACAGTCTCCTAAAGTATCCTCCTAAAGTCACCTCCTAACGTCCATCCTAAAGCCAACACCTAAAGCCTACACCTAAAGACCCATCAAGTCAACGCCTATCTTAAAGTTTAAACATAAAGACCAGACC                                                                                                                                                                                                                                                                                                    <  1:368088/287‑1`  
           `tgtgtataagagacaGGACCTAAAGTT‑CCCCCATG‑GGGGGTACCTAAAGCCCAGCCAATCACCTAAAGTCAACCTTCGGTTGACCTTGAGGGTTCCCTAAGGGTTGGGGATGACCATTGGGTTTGTCTTTGGGTGTTACCTTGAGTGTCTCTCTGTGTCCCTATCTGTTACAGTCTCCTAAAGTATCCTCCTAAAGTCACCTCCTAACGTCCATCCTAAAGCCAACACCTAAAGCCTACACCTAAAGACCCATCAAGTCAACGCCTATCTTAAAGTTTAAACATAAAGACCAGACCTAAAG                                                                                                                                                                                                                                                                                               <  1:415434/286‑1`  
           `ataagagacagGTACGGACCTAAAGTT‑CCCCCATA‑GGGGGTACCTAAAGCCCAGCCAATCACCTAAAGACAACCTTCGGTTGACCTTGAGGGTTCCCTAAGGGTTGGGGATGACCCATGGGTTTGTCTTTGGGTGTTACCTTGAGTGTCTCTCTGTGTCCCTATCTGTTACAGTCTCCTAAAGTATCCTCCTAAAGTCACCTCCTAACGTCCATCCTAAAGCCAACACCTAAAGCCTACACCTAAAGACCCATCAAGTCAACGCCTATCTTAAAGTTTAAACATAAAGACCAGACCTAAAG                                                                                                                                                                                                                                                                                               <  1:330316/290‑1`  
           `aagagacagGTGTACGGACCTAAAGTT‑CCCCCATA‑GGTGGTACCTAAAGCCCAGCCAATCACCTAAAGTCAACCTTCGGTTGACCTTGAGGGTTCCCTAAGGGTTGGGGATGACCCTTGGGTTTGTCTTTGGGTGTTACCTTGAGTGTCTCTCTGTGTCCCTATCTGTTACAGTCTCCTAAAGTATCCTCCTAAAGTCACCTCCTAACGTACATCCTAAAGCCAACACCTAAAGCCTACACCTAAAGACCCATCAAGTCAACGCCTATCTTAAAGTTTAAACATAAAGACCAGACCTAAAG                                                                                                                                                                                                                                                                                               <  1:115629/292‑1`  
           `aagagacagGTGTACGGACCTAAAGTT‑CCCCCATA‑GGGGGTACCTAAAGCCCAGCCAATCACCTAAAGTCAACCTTCGGTTGACCTTGAGGGTTCCCTAAGGGTTGGGGATGACCCTTGGGTTTGTCTTTGGGTGTTACCTTGAGTGTCTCTCTGTGTCCCTATCTGTTACAGTCTCCTAAAGTATCCTCCTAAAGTCACCTCCTAACGTCCATCCTAAAGCCAACACCTAAAGCCTACACCTAAAGACCCATCAAGTCAACGCCTATCTTAAAGTTTAAACATAAAGACCAGACCTAAAG                                                                                                                                                                                                                                                                                               <  1:544899/292‑1`  
           `aagagacagGTGTACGGACCTAAAGTT‑CCCCCATA‑GGGGGTACCTAAAGCCCAGCCAATCACCTAAAGTCAACCTTCGGTTGACCTTGAGGGTTCCCTAAGGGTTGGGGATGACCCTTGGGTTTGTCTTTGGGTGTTACCTTGAGTGTCTCTCTGTGTCCCTATCTGTTACAGTCTCCTAAAGTATCCTCCTAAAGTCACCTCCTAACGTCCATCCTAAAGCCAACACCTAAAGCCTACACCTAAAGACCCATCAAGTCAACGCCTATCTTAAAGTTTAAACATAAAGACCAGACCTAAAG                                                                                                                                                                                                                                                                                               <  1:17046/292‑1`  
           `aagagacagGTGTACGGACCTAAAGTT‑CCCCCATA‑GGGGGTACCTAAAGCCCAGCCAATCACCTAAAGTCAACCTTCGGTTGACCTTGAGGGTTCCCTAAGGGTTGGGGATGACCCTTGGGTTTGTCTTTGGGTGTTACCTTGAGTGTCTCTCTGTGTCCCTATCTGTTACAGTCTCCTAAAGTATCCTCCTAAAGTCACCTCCTAACGTCCATCCTAAAGCCAACACCTAAAGCCTACACCTAAAGACCCATCAAGTCAACGCCTATCTTAAAGTTTAAACATAAAGACCAGACCTAAAG                                                                                                                                                                                                                                                                                               <  1:432705/292‑1`  
           `aagagacagGTGTACGGACCTAAAGTT‑CCCCCATA‑GGGGGTACCTAAAGCCCAGCCAATCACCTAAAGTCAACCTTCGGTTGACCTTGAGGGTTCCCTAAGGGTTGGGGATGACCCTTGGGTTTGTCTTTGGGTGTTACCTTGAGTGTCTCTCTGTGTCCCTATCTGTTACAGTCTCCTAAAGTATCCTCCTAAAGTCACCTCCTAACGTCCATCCTAAAGCCAACACCTAAAGCCTACACCTAAAGACCAATCAAGTCAACGCCTATCTTAAAGTTTAAACATAAAGACCAGACCTAAAG                                                                                                                                                                                                                                                                                               <  1:489230/292‑1`  
             `gacagCAGTGTACGGACCTAAAGTT‑CCCCCATA‑GGGGGTACCTAAAGCCCAGCCAATCACCTAAAGTCAACCTTCGGTTGACCTTGAGGGTTCCCTAAGGGTTGGGGATGACCCTTGGGTTAGTCTTTGGGTGTTACCTTGAGTGTCTCTCTGTGTCCCTATCTGTTACAGTCTCCTAAAGTATCCTCCTAAAGTCACCTCCTAACGTCCATCCTAAAGCCAACACCTAAAGCCTACACCTAAAGACCCATCAAGTCAACGCCTATCTTAAAGTTTAAACATAAAGACCAGACCTAAAGAC                                                                                                                                                                                                                                                                                             <  1:506921/296‑1`  
             `aagagacagggACGGACCTAAAGTT‑CCCCCATA‑GGGGGTACCTAAAGCCCAGCCAATCACCTAAAGTCAACCTTCGGTTGACCTGGAGGGTTCCCTAAGGGTTGGGGATGACCCTTGGGTTTGTCTTTGGGTGTTACCTTGAGTGTCTCTCTGTGTCCCTATCTGTTACAGTCTCCTAAAGTATCCTCCTAAAGTCACCTCCTAACGTCCATCCTAAAGCCAACACCTAAAGCCTACACCTAAAGACCCATCAAGTCAACGCCTATCTTAAAGTTTAAACATAAAGACCAGACCTAAAGAC                                                                                                                                                                                                                                                                                             <  1:323380/290‑1`  
              `gtataagagacaGGACCTAAAGTT‑CCCCCATC‑GGGGGTACCTAAAGCCCAGCCAATCACCTAAAGTCCACCTTCGGTTGACCTTGAGGGTTCCCTAAGGGTTGGGGATGACCCTTGGGTTTGTCTTTGGGTGTTACTTTGAGTGTCTCTCTGTGTCCCTATCTGTTACAGTCTCCTAAAGTATCCTACTAAAGTCACCTCCTAACGTCCATCCTAAAGCCAACACCTAAAGCCTACACCTAAAGACCCATCAAGTCAACGCCTATCTTAAAGTTTAAACATAAAGACCAGACCTAAAGACC                                                                                                                                                                                                                                                                                            <  1:597474/289‑1`  
              `agagacagGTAAGGACCAAAATTT‑CCCCCATA‑GGGGGTACCTAAAGCCCAGCCAATCACCGAAAGTCAACCTTCGGTTGACCTTGAGGGTTCCCTAAGGGTTGGGGATGACCCTTGGGTTTGTCTTGGGGTGTTACCTTGAGTGTCTCTCTGTGTCCCTATCTGTTACAGTCTCCTAAAGTATCCTCCTAAAGTCACCTCCTAACGTCCATCCTAAAGCCAACACCTAAAGCCTACACCTAAAGACCCATCAAGTCAACGCCTATCTTAAAGTTTAAACATAAAGACCAGACCTAAAGACC                                                                                                                                                                                                                                                                                            <  1:647096/293‑1`  
              `agacagGTGTACGTACCTATAGTT‑CCCACATA‑GGTGTTACCTAAAGCCCAGCCAATCACCTAAAGTCAACCTTCGGTTTACCTTGATGGTTCCCTAAGGGTTTGGGATGACCCTTGGGTTTGTCTCTGGGTGTTACCTTGAGTTTCTCTCTGTGTCCCTATCTGTTACAGTCTCCTCAAGTATCCTCCTAAAGACACCTCCTAACGTCCATCCTAAAGCCAACACCTAAAGTCTACACCTAAAGACCCATCAAGTCAACGCCTATCTAAAAGTTTAAACATAAGGACCAGACCTAAAGACC                                                                                                                                                                                                                                                                                            <  1:668839/295‑1`  
              `agacagGTGTACGGACCTAAAGTT‑CCCCCATA‑GGGGGTACCTAAAGCCCAGCCAATCACCTAAAGTCAACCTTCGGTTGACCTTGAGGGTTCCCTAAGGGTTGGGGATGACCCTTGGGTTTGTCTTTGGGTGTTACCTTGAGTGTCTCTCTGTGTCCCTATCTGTTACAGTCTCCTAAAGTATCCTCCTAAAGTCACCTCCTAACGTCCATCCTAAAGCCAACACCTAAAGCCTACACCTAAAGACCCATCAAGTCAACGCCTATCTTAAAGTTTAAACATAAAGACCAGACCTAAAGACC                                                                                                                                                                                                                                                                                            <  1:100028/295‑1`  
                   `cagGTACGGACCTAAAGTT‑CCCCCCTA‑GGGTGTACCTAAAGCCCAGCCTATCACCTAACGTCAACCTTCGGTTGACCTTGAGGGTTCCCTAAGGGTTGGGGATGACCCTTGGGTTTGTCTTGGGGTGTTACCTTGAGTGTCTCTCTGTGTCCCCATCTGTTACAGTCTCCTAAAGTATCCTCCTAAAGTCACCTCCTAACGTCCATCCTAAAGCCAACACCTAAAGCCTACACCTAAAGACCCATCAAGTCAACGCCTATCTTAAAGTTTAAACATAAAGACCAGACCTAAAGACCAGACC                                                                                                                                                                                                                                                                                       <  1:560421/298‑1`  
                    `aagatacagCCTAAAGTT‑CCCCCATA‑GGGGGGACCTAAAGCCCAGCCAATCACCTAAAGTCAACCTTCGGTTGACCTTGAGGGTTCCCTAAGGGTTGGGGATGACACTTGGGTTTGTCTTTGGGTGTTACCTTGAGTGTCTCTCTGTGTCCCTATCTGTTACAGTCTCCTAAAGTATCCTCCTAAAGTCACCTCCTAACGTCCATCCTAAAGCCAACACCTAAAGCCTACACCTAAAGACCCATCAAGTCAACGCCTATCTTAAAGTTTAAACATAAAGACCAGACCTAAAGACCAGACCT                                                                                                                                                                                                                                                                                      <  1:384005/292‑1`  
                    `GTGTACGTACCTAAAGTT‑CCCCCATA‑GTGGGTACCTAAAGCCAAGCCAATCACCTAAAGTCAACCTTCGGTTGACCTTGAGGATTCCCTAAGGGTTGGGGATGACCCTTGGGTTTGTCTTTGGTTGTTACCTTGAGTGTCTCTCTGTGTCCCTTTCTGTTACAGTCTCCTAAAGTATCCTACTAAAGTCACCTCCTAACGTACAACCTAAAGCAAACACCTAAAGCCTACACCTCAATACCAATCAAGTCAACCCCTATCTTAAAGTTTAAACCTAAAAACCAAACCTAAATACCAAACCT                                                                                                                                                                                                                                                                                      >  1:87690/1‑301`  
                    `GTGTACGGCCCTAAAGTT‑CCCCCATA‑GGTGGTACCTAAAGCCCAGCCAATCACCTAAAGTCAACCTTCGGTTGACCTTGAGGGTTCCCTAAGGGTTGGGGATGACCCTTGGGTTTGTCTTTGGGTGTTACCTTGAGTGTCTCTCTGTGTCCCTATCTGTTACAGTCTCCTAAAGTATCCTCCTAAAGTCACCTCCTAACGTCCATCCTAAAGCCAACACCTAAAGCCTACACCTAAAGACCCATCAAGTCAACGCCTATCTACAAGTTTAAACATAAAGACCAGACCTAAAGACCAGACCT                                                                                                                                                                                                                                                                                      >  1:335368/1‑301`  
                    `GTGTACGGCCCTAAAGTT‑CCCCCATA‑GGGGGTACCTAAAGCCCAGCCAATCACCTAAAGTCAACCTTCGGTTGACCTTGAGGGTTCCCTAAGGGTTGGGGATGACCCTTGGGTTTGTCTTTGGGTGTTACCTTGAGTTTCTCTCTGTGTCCCTATCTGTTACAGTCTCCTAAAGTATCCTCCTAAAGTCACCTCCTAACGTCCATCCTAAAGCCAACACCTAAAGCCTACACCTAAAGACCCATCAAGTCAACGCCTATCTTAAAGTTTAAACATAAAGACCAAACCctgtctcttataca                                                                                                                                                                                                                                                                                      >  1:423086/1‑287`  
                    `GTGTACGGACCTAAAGTT‑TCCCCATA‑GGGGGTACCTAAAGCCCAGCCAATCACCTAAAGTCAACCTTCGGTTGACCTTGAGGGTTCCCTAAGGGTTGGGGATGACCCTTGGGTTTGTCTTTGGGTGTTACCTTGAGTGTCTCTCTGTGTCCCTATCTGTTACAGTCTCCTAAAGTATCCTCCTAAAGTCACCTCCTAACGTCCATCCTAAAGCCAACACCTAAAGCCTACACCTAAAGACCCATCAAGTCAAAGCCTATCTTAAAGTTTAAACATAAAGACCAGACCTAAAGACCAGACCa                                                                                                                                                                                                                                                                                      >  1:371556/1‑300`  
                    `GTGTACGGACCTAAAGTT‑CCCCCATA‑TGGGGTACCTAAAGCCCAGCCAATCACCTAAAGTCAACCTTCGGTTGACCTTGAGGGTTCCCTAAGGGTTGGGGATGACCCTTGGGTTTGTCTTTGGGTGTTACCTTGAGTGTCTCTCTGTGTCCCTATCTGTTACAGTCTCCTAAAGTATCCTCCTAAAGTCACCTCCTAACGTCCATCCTAAAGCCAACACCTAAAGCCTACACCTAAAGACCCATCAAGTCAACGCCTATCTTAAAGTTTAAACATAAAGACCAGACCTAAAGACCAGACCT                                                                                                                                                                                                                                                                                      >  1:404317/1‑301`  
                    `GTGTACGGACCTAAAGTT‑CCCCCATA‑GGTGGTACCTAAAGCCCAGCCAATCACCTAAAGTCAACCTTCGGTTGACCTTGAGGGTTCCCTAAGGGTTGGGGATGACCCTTGGGTTTGTCTTTGGGTGTTACCTTGAGTATCTCTCTGTGTCCCTATCTGTTACAGTCTGCTAAAGTATCCTCCTAAAGTCACCTCCTAACGTCCATCCTAAAGCCAACACCTAAAGCCTACACCTAAAGACCCATCAAGTCAACGCCTATCTTAAAGTTTAAACATAAAGACCAGACCTAAAGACAAGACCT                                                                                                                                                                                                                                                                                      >  1:293650/1‑301`  
                    `GTGTACGGACCTAAAGTT‑CCCCCATA‑GGGGGTACCTAAAGCCCAGCCAATCACCTAAAGTCAACCTTCGGTTGACCTTGAGGGTTTCCTAAGGGTTGGGGATGACCCTTGGGTTTGTCTTTGGGTGTTACCTTGAGTGTCTCTCTGTGTCCCTATCTGTTACAGTCTCCTAAAGTATCCTCCTAAAGTCACCTCCTAACGTCCATCCTAAAGCCAACACCTAAAGCCTACACCTAAAGACCCATCAAGTCAACGCCTATCTTAAAGTTTAAACATAAAGACCctgtctcttatacacatct                                                                                                                                                                                                                                                                                      >  1:143882/1‑282`  
                    `GTGTACGGACCTAAAGTT‑CCCCCATA‑GGGGGTACCTAAAGCCCAGCCAATCACCTAAAGTCAACCTTCGGTTGACCTTGAGGGTTCCCTAAGGGTTGGGGATGACCCTTGGGTTTGTCTTTGGGTGTTATCTTGAGTGTCTCTCTGTGTCCCTATCTGTTACAGTCTCCTAAAGTATCCTCCTAAAGTCACCTCCTAACGTCCATCCTAAAGCCAACACCTAAAGCCTACACCTAAAGACCCATCAAGTAAACGCCTATCTTAAAGTTTAAACATAAAGACCAGACCctgtctcttataca                                                                                                                                                                                                                                                                                      >  1:277354/1‑287`  
                    `GTGTACGGACCTAAAGTT‑CCCCCATA‑GGGGGTACCTAAAGCCCAGCCAATCACCTAAAGTCAACCTTCGGTTGACCTTGAGGGTTCCCTAAGGGTTGGGGATGACCCTTGGGTTTGTCTTTGGGTGTTACCTTGAGTGTCTCTCTGTGTCCCTATCTGTTACAGTCTTCTAAAGTATCCTCCTAACGTCACTTCCTAACGTCCATCCTAAAGCCAACACCTAAAGCCTACACCAAAAGACCAATCAAGTCAACGCCTATTTTAACGTTTAAACATAAAGACCAGACCTAAAGACCAGACCT                                                                                                                                                                                                                                                                                      >  1:438045/1‑301`  
                    `GTGTACGGACCTAAAGTT‑CCCCCATA‑GGGGGTACCTAAAGCCCAGCCAATCACCTAAAGTCAACCTTCGGTTGACCTTGAGGGTTCCCTAAGGGTTGGGGATGACCCTTGGGTTTGTCTTTGGGTGTTACCTTGAGTGTCTCTCTGTGTCCCTATCTGTTACAGTCTCCTAAAGTATCCTCCTAAAGTCACCTCCTAACGTCCCTCCTAAAGCCAACACCTAACGCCTACACCTAAAGACCCATCAAGTCAACGCCTATCTTAAAGTTTAAACATAAAGACCAGACCTAAAGACGAGACCT                                                                                                                                                                                                                                                                                      >  1:555601/1‑301`  
                    `GTGTACGGACCTAAAGTT‑CCCCCATA‑GGGGGTACCTAAAGCCCAGCCAATCACCTAAAGTCAACCTTCGGTTGACCTTGAGGGTTCCCTAAGGGTTGGGGATGACCCTTGGGTTTGTCTTTGGGTGTTACCTTGAGTGTCTCTCTGTGTCCCTATCTGTTACAGTCTCCTAAAGTATCCTCCTAAAGTCACCTCCTAACGTCCATCCTAAAGCCGACACCTCAAGCCTACACCTAAAGACCCATCAAGTCAACGCCTATCTTAAAGTTTCCACATAAAGACCAGACCTAAAGACCAGACCT                                                                                                                                                                                                                                                                                      >  1:640011/1‑301`  
                    `GTGTACGGACCTAAAGTT‑CCCCCATA‑GGGGGTACCTAAAGCCCAGCCAATCACCTAAAGTCAACCTTCGGTTGACCTTGAGGGTTCCCTAAGGGTTGGGGATGACCCTTGGGTTTGTCTTTGGGTGTTACCTTGAGTGTCTCTCTGTGTCCCTATCTGTTACAGTCTCCTAAAGTATCCTCCTAAAGTCACCTCCTAACGTCCATCCTAAAGCCAACACCTAAAGCCTACACCTAACGACCCATCAAGTCAACGCCAATCTTAAAGTTTAAACATAAAGACCAAACCTAAAGACCAAACCT                                                                                                                                                                                                                                                                                      >  1:550447/1‑301`  
                    `GTGTACGGACCTAAAGTT‑CCCCCATA‑GGGGGTACCTAAAGCCCAGCCAATCACCTAAAGTCAACCTTCGGTTGACCTTGAGGGTTCCCTAAGGGTTGGGGATGACCCTTGGGTTTGTCTTTGGGTGTTACCTTGAGTGTCTCTCTGTGTCCCTATCTGTTACAGTCTCCTAAAGTATCCTCCTAAAGTCACCTCCTAACGTCCATCCTAAAGCCAACACCTAAAGCCTACACCTAAAGACCCATCAAGTCAACGCCTATCTTAAAGTTTAAACATAAAGACCAGACCTAACGACCAGACCT                                                                                                                                                                                                                                                                                      >  1:12911/1‑301`  
                    `GTGTACGGACCTAAAGTT‑CCCCCATA‑GGGGGTACCTAAAGCCCAGCCAATCACCTAAAGTCAACCTTCGGTTGACCTTGAGGGTTCCCTAAGGGTTGGGGATGACCCTTGGGTTTGTCTTTGGGTGTTACCTTGAGTGTCTCTCTGTGTCCCTATCTGTTACAGTCTCCTAAAGTATCCTCCTAAAGTCACCTCCTAACGTCCATCCTAAAGCCAACACCTAAAGCCTACACCTAAAGACCCATCAAGTCAACGCCTATCTTAAAGTTTAAACATAAAGACCAGACCTAAAGctgtctctt                                                                                                                                                                                                                                                                                      >  1:207883/1‑292`  
                    `GTGTACGGACCTAAAGTT‑CCCCCATA‑GGGGGTACCTAAAGCCCAGCCAATCACCTAAAGTCAACCTTCGGTTGACCTTGAGGGTTCCCTAAGGGTTGGGGATGACCCTTGGGTTTGTCTTTGGGTGTTACCTTGAGTGTCTCTCTGTGTCCCTATCTGTTACAGTCTCCTAAAGTATCCTCCTAAAGTCACCTCCTAACGTCCATCCTAAAGCCAACACCTAAAGCCTACACCTAAAGACCCATCAAGTCAACGCCTATCTTAAAGTTTAAACATAAAGACCAGACCTAAAGACCtgtctc                                                                                                                                                                                                                                                                                      >  1:243320/1‑295`  
                    `GTGTACGGACCTAAAGTT‑CCCCCATA‑GGGGGTACCTAAAGCCCAGCCAATCACCTAAAGTCAACCTTCGGTTGACCTTGAGGGTTCCCTAAGGGTTGGGGATGACCCTTGGGTTTGTCTTTGGGTGTTACCTTGAGTGTCTCTCTGTGTCCCTATCTGTTACAGTCTCCTAAAGTATCCTCCTAAAGTCACCTCCTAACGTCCATCCTAAAGCCAACACCTAAAGCCTACACCTAAAGACCCATCAAGTCAACGCCTATCTTAAAGTTTAAACATAAAGACCAGACCTAAAGACCAGACCT                                                                                                                                                                                                                                                                                      >  1:230585/1‑301`  
                    `GTGTACGGACCTAAAGTT‑CCCCCATA‑GGGGGTACCTAAAGCCCAGCCAATCACCTAAAGTCAACCTTCGGTTGACCTTGAGGGTTCCCTAAGGGTTGGGGATGACCCTTGGGTTTGTCTTTGGGTGTTACCTTGAGTGTCTCTCTGTGTCCCTATCTGTTACAGTCTCCTAAAGTATCCTCCTAAAGTCACCTCCTAACGTCCATCCTAAAGCCAACACCTAAAGCCTACACCTAAAGACCCATCAAGTCAACGCCTATCTTAAAGTTTAAACATAAAGACCAGACCTAAAGACCAGACCT                                                                                                                                                                                                                                                                                      >  1:28936/1‑301`  
                    `GTGTACGGACCTAAAGTT‑CCCCCATA‑GGGGGTACCTAAAGCCCAGCCAATCACCTAAAGTCAACCTTCGGTTGACCTTGAGGGTTCCCTAAGGGTTGGGGATGACCCTTGGGTTTGTCTTTGGGTGTTACCTTGAGTGTCTCTCTGTGTCCCTATCTGTTACAGTCTCCTAAAGTATCCTCCTAAAGTCACCTCCTAACGTCCATCCTAAAGCCAACACCTAAAGCCTACACCTAAAGACCCATCAAGTCAACGCCTATCTTAAAGTTTAAACATAAAGACCAGACCTAAAGACCAGACCT                                                                                                                                                                                                                                                                                      >  1:163454/1‑301`  
                    `GTGTACGGACCTAAAGTT‑CCCCCATA‑GGGGGTACCTAAAGCCCAGCCAATCACCTAAAGTCAACCTTCGGTTGACCTTGAGGGTTCCCTAAGGGTTGGGGATGACCCTTGGGTTTGTCTTTGGGTGTTACCTTGAGTGTCTCTCTGTGTCCCTATCTGTTACAGTCTCCTAAAGTATCCTCCTAAAGTCACCTCCTAACGTCCATCCTAAAGCCAACACCTAAAGCCTACACCTAAAGACCCATCAAGTCAACGCCTATCTTAAAGTTTAAACATAAAGACCAGACCTAAAGACCAGACCT                                                                                                                                                                                                                                                                                      >  1:478449/1‑301`  
                    `GTGTACGGACCTAAAGTT‑CCCCCATA‑GGGGGTACCTAAAGCCCAGCCAATCACCTAAAGTCAACCTTCGGTTGACCTTGAGGGTTCCCTAAGGGTTGGGGATGACCCTTGGGTTTGTCTTTGGGTGTTACCTTGAGTGTCTCTCTGTGTCCCTATCTGTTACAGTCTCCTAAAGTATCCTCCTAAAGTCACCTCCTAACGTCCATCCTAAAGCCAACACCTAAAGCCTACACCTAAAGACCCATCAAGTCAACGCCTATCTTAAAGTTTAAACATAAAGACCAGACCTAAAGACCAGACCT                                                                                                                                                                                                                                                                                      >  1:184945/1‑301`  
                    `GTGTACGGACCTAAAGTT‑CCCCCATA‑GGGGGTACCTAAAGCCCAGCCAATCACCTAAAGTCAACCTTCGGTTGACCTTGAGGGTTCCCTAAGGGTTGGGGATGACCCTTGGGTTTGTCTTTGGGTGTTACCTTGAGTGTCTCTCTGTGTCCCTATCTGTTACAGTCTCCTAAAGTATCCTCCTAAAGTCACCTCCTAACGTCCATCCTAAAGCCAACACCTAAAGCCTACACCTAAAGACCCATCAAGTCAACGCCTATCTTAAAGTTTAAACATAAAGACCAGACCTAAAGACCAGACCT                                                                                                                                                                                                                                                                                      >  1:247012/1‑301`  
                    `GTGTACGGACCTAAAGTT‑CCCCCATA‑GGGGGTACCTAAAGCCCAGCCAATCACCTAAAGTCAACCTTCGGTTGACCTTGAGGGTTCCCTAAGGGTTGGGGATGACCCTTGGGTTTGTCTTTGGGTGTTACCTTGAGTGTCTCTCTGTGTCCCTATCTGTTACAGTCTCCTAAAGTATCCTCCTAAAGTCACCTCCTAACGTCCATCCTAAAGCCAACACCTAAAGCCTACACCTAAAGACCCATCAAGTCAACGCCTATCTTAAAGTTTAAACATAAAGACCAGACCTAAAGACCAGACCT                                                                                                                                                                                                                                                                                      >  1:52258/1‑301`  
                    `GTGTACGGACCTAAAGTT‑CCCCCATA‑GGGGGTACCTAAAGCCCAGCCAATCACCTAAAGTCAACCTTCGGTTGACCTTGAGGGTTCCCTAAGGGTTGGGGATGACCCTTGGGTTTGTCTTTGGGTGTTACCTTGAGTGTCTCTCTGTGTCCCTATCTGTTACAGTCTCCTAAAGTATCCTCCTAAAGTCACCTCCTAACGTCCATCCTAAAGCCAACACCTAAAGCCTACACCTAAAGACCCATCAAGTCAACGCCTATCTTAAAGTTTAAACATAAAGACCAGACCTAAAGACAAGACCT                                                                                                                                                                                                                                                                                      >  1:574550/1‑301`  
                    `GTGTACGGACCTAAAGTT‑CCCCCATA‑GGGGGTACCTAAAGCCCAGCCAATCACCTAAAGTCAACCTTCGGTTGACCTTGAGGGTTCCCTAAGGGTTGGGGATGACCCTTGGGTTTGTCTTTGGGTGTTACCTTGAGTGTCTCTCTGTGTCCCTATCTGTTACAGTCTCCTAAAGTATCCTCCTAAAGTCACCTCCTAACGTCCATCCTAAAGCCAACACCTAAAGCCTACACCTAAAGACCCATCAAGTCAACGCCTATCTTAAAGTTTAAACATAAAGACCAGAACTAAAGACCctgtcc                                                                                                                                                                                                                                                                                      >  1:459847/1‑295`  
                    `GTGTACGGACCTAAAGTT‑CCCCCATA‑GGGGGTACCTAAAGCCCAGCCAATCACCTAAAGTCAACCTTCGGTTGACCTTGAGGGTTCCCTAAGGGTTGGGGATGACCCTTGGGTTTGTCTTTGGGTGTTACCTTGAGTGTCTCTCTGTGTCCCTATCTGTTACAGTCTCCTAAAGTATCCTCCTAAAGTCACCTCCTAACGTCCATCCTAAAGCCAACACCTAAAGCCTACACCTAAAGACCCATCAAGTCAACGCCTATCTTAAAGTTTAAACATAAAGAACAGACCTAAAGACCAGACCT                                                                                                                                                                                                                                                                                      >  1:526016/1‑301`  
                    `GTGTACGGACCTAAAGTT‑CCCCCATA‑GGGGGTACCTAAAGCCCAGCCAATCACCTAAAGTCAACCTTCGGTTGACCTTGAGGGTTCCCTAAGGGTTGGGGATGACCCTTGGGTTTGTCTTTGGGTGTTACCTTGAGTGTCTCTCTGTGTCCCTATCTGTTACAGTCTCCTAAAGTATCCTCCTAAAGTCACCTCCTAACGTCCATCCTAAAGCCAACACCTAAAGCCTACACCTAAAGACCCATCAAGTCAACGCCTATCTTAAAGTTTAAAAATAAAGACCAGACCTAAAGACCAGAACT                                                                                                                                                                                                                                                                                      >  1:219999/1‑301`  
                    `GTGTACGGACCTAAAGTT‑CCCCCATA‑GGGGGTACCTAAAGCCCAGCCAATCACCTAAAGTCAACCTTCGGTTGACCTTGAGGGTTCCCTAAGGGTTGGGGATGACCCTTGGGTTTGTCTTTGGGTGTTACCTTGAGTGTCTCTCTGTGTCCCTATCTGTTACAGTCTCCTAAAGTATCCTCCTAAAGTCACCTCCTAACGTCCATCCTAAAGCCAACACCTAAAGCCTACACCTAAAGACCCATCAAGTCAACGCCTATCGTAAAGTTTAAACATAAAGACCAGACCTAAAGACCAGACCT                                                                                                                                                                                                                                                                                      >  1:515105/1‑301`  
                    `GTGTACGGACCTAAAGTT‑CCCCCATA‑GGGGGTACCTAAAGCCCAGCCAATCACCTAAAGTCAACCTTCGGTTGACCTTGAGGGTTCCCTAAGGGTTGGGGATGACCCTTGGGTTTGTCTTTGGGTGTTACCTTGAGTGTCTCTCTGTGTCCCTATCTGTTACAGTCTCCTAAAGTATCCTCCTAAAGTCACCTCCTAACGTCCATCCTAAAGCCAACACCTAAAGCCTACACCTAAAGACCCATCAAGTCAACGACTATCTTAAAGTTTAAACATAAAGACCAGACCTAAAGctgtctctt                                                                                                                                                                                                                                                                                      >  1:593204/1‑292`  
                    `GTGTACGGACCTAAAGTT‑CCCCCATA‑GGGGGTACCTAAAGCCCAGCCAATCACCTAAAGTCAACCTTCGGTTGACCTTGAGGGTTCCCTAAGGGTTGGGGATGACCCTTGGGTTTGTCTTTGGGTGTTACCTTGAGTGTCTCTCTGTGTCCCTATCTGTTACAGTCTCCTAAAGTATCCTCCTAAAGTCACCTCCTAACGTCCATCCTAAAGCCAACACCTAAAGCCTACACCTAAAGACACATCAAGTCAATGCCTATCTTAAAGTTTAAACATAAAGACCAGCACTAAAGACCAGCCCc                                                                                                                                                                                                                                                                                      >  1:49576/1‑300`  
                    `GTGTACGGACCTAAAGTT‑CCCCCATA‑GGGGGTACCTAAAGCCCAGCCAATCACCTAAAGTCAACCTTCGGTTGACCTTGAGGGTTCCCTAAGGGTTGGGGATGACCCTTGGGTTTGTCTTTGGGTGTTACCTTGAGTGTCTCTCTGTGTCCCTATCTGTTACAGTCTCCTAAAGTATCATCCTAAAGTCACCTCCTAACGTCCATCCTAAAGCCACCACCTAAAGCCTACACCTAAAGACCCATCAACTCAACGCCTATCTTAAAGTTTAAACATAAAGACCAGAACTAAAGACAAGACCT                                                                                                                                                                                                                                                                                      >  1:447466/1‑301`  
                    `GTGTACGGACCTAAAGTT‑CCCCCATA‑GGGGGTACCTAAAGCCCAGCCAATCACCTAAAGTCAACCTTCGGTTGACCTTGAGGGTTCCCTAAGGGTTGGGGATGACCCTTGGGTTTGTCTTTGGGTGTTACCTTGAGTGTCTCTCTGTGTCCCGATCGGTTACAGTCTCCTAAAGTATCCTCCTAAAGTCACCTCCTAACGTCCATCCTAAAGCCAACACCTAAAGCCTACACCTAAAGAACCATCAAGTCAACGCCTAACTTAAAGTTTAAAACTAAAGACAAGACCTAAAGACCACACCT                                                                                                                                                                                                                                                                                      >  1:631028/1‑301`  
                    `GTGTACGGACCTAAAGTT‑CCCCCATA‑GGGGGTACCTAAAGCCCAGCCAATCACCTAAAGTCAACCTTCGGTTGACCTTGAGGGTTCCCTAAGGGTTGGGGATGACCCTTGGGTTTGTCTTTGGGTGTTACCTTGAGTGTCTCTCTGTGTACCTATCTGTTACAGTCTCCTAAAGTATCCTCCTAAAGTCACCTCCTAACGTCCATCCTAAAGCCAACACCTAAAGCCTACACCTAAAGACCCATCAAGTCAACGCCTATCTTAAAGTTTAAACATAAAGACCAGACCTAAAGACCAGACaa                                                                                                                                                                                                                                                                                      >  1:80378/1‑299`  
                    `GTGTACGGACCTAAAGTT‑CCCCCATA‑GGGGGTACCTAAAGCCCAGCCAATCACCTAAAGTCAACCTTCGGTTGACCTTGAGGGTTCCCTAAGGGTTGGGGATGACCCTTGGGTTTGTCTTTGGGTGTTACCTTGAGTGTCTCTATGTGTCCCTATCTGTTACAGTCTCCTAAAGTATCCTCCTAAAGTCACCTCCTAACGTCCATCCTAAAGCCAACACCTAAAGCCTACACCTAAAGACCCATCAAGTCAACGCCTATCTTAAAGTTTAAACATAAAGACCtgtctcttatacacatctc                                                                                                                                                                                                                                                                                      >  1:567884/1‑282`  
                    `GTGTACGGACCTAAAGTT‑CCCCCATA‑GGGGGTACCTAAAGCCCAGCCAATCACCTAAAGTCAACCTTCGGTTGACCTTGAGGGTTCCCTAAGGGTTGGGGATGACCCTTGGGTTTATCTTTGGGTGTTACCTTGAGTGTCTCTCTGTGTCCCTATCTGTTACAGTCTCCTAAAGTATCCTCCTAAAGTCACCTCCTAACGTCCATCCTAAAGCCAACACCTAAAGCCTACACCTAAAGACCCATCAAGTCAACGCCTATCTTAAAGTTTAAACATAAAGACCAGACCTAAAGACCAGACCT                                                                                                                                                                                                                                                                                      >  1:31943/1‑301`  
                    `GTGTACGGACCTAAAGTT‑CCCCCATA‑GGGGGTACCTAAAGCCCAGCCAATCACCTAAAGTCAACCTTCGGTTGACCTTGAGGGTTCCCTAAGGGTTGGGGATGACCCTTGGGGTTGTCTTTGGGTGTTACCTTGAGTGTCTCTCTGTGTCCCTATCTGTTACAGTCTCCTAAAGTATCCTCCTAAAGTCACCTCCTAACGTCCATCCTAACGCCAACACCTAAAGCCTACACCTAAAGACCCATCACGTCAACACCTATCTTAGAGTTTAAACATAAAGACCCGACCTCAAacactgtctc                                                                                                                                                                                                                                                                                      >  1:396081/1‑291`  
                    `GTGTACGGACCTAAAGTT‑CCCCCATA‑GGGGGTACCTAAAGCCCAGCCAATCACCTAAAGTCAACCTTCGGTTGACATTGAGGGTTCCCTAAGGGTTGGGGATGACCCTTGGGTTTGTCTTTGGGTGTTACCTTGAGTGTCTCTCTGTGTCCCTATCTGTTACAGTCTACTAAAGTATCCTCCTAAAGTCACCTCCTAACGTCCATCCTAAAGCCAACACCTAAAGCCTACACCTAAAGACCCATCAAGTCAACGCCTATCTTAAAGTTTAAACATAAAGACCAGACCTAAAGACCAGACCT                                                                                                                                                                                                                                                                                      >  1:266963/1‑301`  
                    `GTGTACGGACCTAAAGTT‑CCCCCATA‑GGGGGTACCTAAAGCCCAGCCAATCACCTAAAGTCAACCTTCGCTTCACCTTGAGGGTTCCCTAATGGTTGGGCTTTACCCTTGGGTTTGTCTTTGGGTGTTTCCTTTAGTGTCTCTCTTTCTCCCTATCTGTTCCAGTCTCCTAATGTATCCTCCTCATGTCACCTACTAACGTCCCTCCTAACGCCAACACCTAAAGCCTTCCCCTAAAGACCCATCAAGTCAACTCCTATCTTAAAGTTTACACTTAAACACCAAACCTAAcgcacacttct                                                                                                                                                                                                                                                                                      >  1:578061/1‑290`  
                    `GTGTACGGACCTAAAGTT‑CCCCCACA‑GGGGGTACCTAAAGCCCAGCCAAGCACCTAAAGTCAACCTTCGGTTGACCTTGAGGGTTCCCTAAGGGTTGGGGATGACCCTTGGGTTTGTCTTTGGGTGTTACCTTGAGTGTCTCTCTGTGTCCCTATCTGTTACAGTCTCCTAAAGTATCCTCCTAAAGTCACCTCCTAACGTCCATCCTAAAGCCAACACCTAAAGCCTACACCTAAATACCAATCAAGTCACCGCCTATCTTAAAGTTTAAACATAAAGACCACACCTAAAGACCAGACCT                                                                                                                                                                                                                                                                                      >  1:179139/1‑301`  
                      `GTACGGACCTAAAGTTCCCCCCATA‑GGGGGTACCTAAAGCCCAGCCAATCACCTAAAGTCAACCTTCGGTTGACCTTGAGGGTTCCCTAAGGGTTGGGGATGACCCTTTGGTTTGTCTTTGGGTGTTACCTTGAGTGTCTCTCTGTGTCCCTATCTGTTACAGTCTCCTAAAGTATCCTCCTAAAGTCACCTCCTAACGTCCATCCTAAAGCTAACACCTAAATCCTACACCTAAAGCCCCATCAAGTCAACGCCTATCTTAAAGTTTAAACATACAGAACATACCTAAAGACCAGACCTA                                                                                                                                                                                                                                                                                     >  1:615961/1‑301`  
                      `GTACGGACCTAAAGTT‑CCCCCATA‑GGGGGTACCTAAAGCCCAGCCAATCACCTAAAGTCAATCTTCGGTTGACCTCGAGGGTTCCCTAAGGGTTGGGGACGACCTTTTGGTTCGTTTTTGGGTGTTACCTTGAGTGTCCCTCTGTGTACCTATCTGTTACAGTCTCTTAAAGTATCCCCCTAAAGTCACCTCCTAACGTCCATCCTAAAGCCAACACCTAAAGCCTACACCTAAAGTCCCATCAATCCAACGCCTACCTCAAAGTTTAAACATAAAGACCAGACTTAAATACCAGAaccaa                                                                                                                                                                                                                                                                                    >  1:110884/1‑296`  
                      `GTACGGACCTAAAGTT‑CCCCCATA‑GGGGGTACCTAAAGCCCAGCCAATCACCTAAAGTCAACCTTCGGTTGACCTTGAGGGTTCCCTAAGGGTTGGGGATGACCCTTGGGTTTGTCTTTGGGTGTTACCTTGAGTGTCTCTCTGTGTCCCTATCTGTTACAGTCTCCTAAAGTATCCTCCTAAAGTCACCTCCTAACGTCCATCCTAAAGCCAACACCTAAAGCCTACACCTAAAGACCCATCAAGTCAACGCCTATCTTAAAGTTTAAACATAAAGACCAGACCTACAGACCAGACCTAA                                                                                                                                                                                                                                                                                    >  1:536289/1‑301`  
                      `GTACGGACCTAAAGTT‑CCCCCATA‑GGGGGTACCTAAAGCCCAGCCAATCACCTAAAGTCAACCTTCGGTTGACCTTGAGGGTTCCCTAAGGGTTGGGGATGACCCTTGGGTTTGTCTTTGGGTGTTACCTTGAGTGTCTCTCTGTGTCCCTATCTGTTACAGTCTCCTAAAGTATCCTCCTAAAGTCACCTCCTAACGTCCATCCTAAAGCCAACACCTAAAGCCTACACCTAAAGACCCATCAAGTCAACGCCTATCTTAAAGTTTAAACATAAAGACCAGACCTAAAGACCctgtctct                                                                                                                                                                                                                                                                                    >  1:530823/1‑293`  
                      `GTACGGACCTAAAGTT‑CCCCCATA‑GGGGGTACCTAAAGCCCAGCCAATCACCTAAAGTCAACCTTCGGTTGACCTTGAGGGTTCCCTAAGGGTTGGGGATGACCCTTGGGTTTGTCTTTGGGTGTTACCTTGAGTGTCTCTCTGTGTCCCTATCTGTTACAGTCTCCTAAAGTATCCTCCTAAAGTCACCTCCTAACGTCCATCCTAAAGCCAACACCTAAAGCCTACACCTAAAGACCCATCAAGTCAACGCCTATCTTAAAGTTTAAACATAAAGACCAGACCTAAAGACCAGACCTAA                                                                                                                                                                                                                                                                                    >  1:252182/1‑301`  
                      `GTACGGACCTAAAGTT‑CCCCCATA‑GGGGGTACCTAAAGCCCAGCCAATCACCTAAAGTCAACCTTCGGTTGACCTTGAGGGTTCCCTAAGGGTTGGGGATGACCCTTGGGTTTGTCTTTGGGTGTTACCTTGAGTGTCTCTCTGTGTCCCTATCTGTTACAGTCTCCTAAAGTATCCTCCTAAAGTCACCTCCTAACGTCCATCCTAAAGCCAACAACTAAAGCCTACACCTAAAGACCCATCAAGTCAACGCCTATCTTAAAGTTCAAACATAAAGACCAGACCTAAAGACCctgtctct                                                                                                                                                                                                                                                                                    >  1:584174/1‑293`  
                      `GTACGGACCTAAAGTT‑CCCCCATA‑GGGGGTACCTAAAGCCCAGCCAATCACCTAAAGTCAACCTTCGGTTGACCTTGAGGGTTCCCTAAGGGTTGGGGATGACCCTTGGGTTTGTCTTTGGGTGTTACCTTGAGTGTCTCTCTGTGTCCCTATCTGTTACAGTCTCCTAAAGTATCCTCCTAAAGTCACCTCCTAACGTCAATCCTAAAGCCAACACCTAAAGCCTACACCTAAAGACCCATCAAGTCAACGCCTATCTTAAAGTTTAAACATAAAGAactgtctcttatacatatctccg                                                                                                                                                                                                                                                                                    >  1:239326/1‑278`  
                      `GTACGGACCTAAAGTT‑CCCCCATA‑GGGGGTACCTAAAGCCCAGCCAATCACCTAAAGTCAACCTTCGGTTGACCTTGAGGGTTCCCTAAGGGTTGGGGATGACCCTTGGGTTTGTCTTTGGGTGTTAACTTGAGTGTCTCTCTGTGTCCCTATCTGTTACAGTCTCCTAAAGTATCCTCCTAAAGTCACATCCTAACGTCCATCCTAAAGCCAACACCTAAAGCCTACACCTAAAGACCCATCAAGTCAACGCCTATCTTAAAGTTTAAACATAAAGACCAGACCTAAAGctgtctattaa                                                                                                                                                                                                                                                                                    >  1:72459/1‑290`  
                      `GTACGGACCTAAAGTT‑CCCCCATA‑GGGGGTACCTAAAGCCCAGCCAATCACCTAAAGTCAACCTTCGGTTGACCTTGAGGGTTCCCTAAGGGTTGGGGATGACCCTTGGGTTTGTATTTGGGTGTTACCTTGAGTGTCTCTCTGTGTCCCTATCTGTTACAGTCTCCCAAAGTATCCTCCTAAAGTCACCTCCTAACGTCCATCCTAAAGCCAACACCTAAAGCCTACACCTAAAGACCCATCAAGTCAACGCCTATCTTAAAGTTTAAACATAAAGACCAGACCTAAAGACCAGACCTgt                                                                                                                                                                                                                                                                                    >  1:274226/1‑299`  
                      `GTACGGACCTAAAGTT‑CCCCCATA‑GGGGGTACATAAAGCCCAGCCAATCACCTAAAGTCAACCTTCGGTTGACCTTGAGGGTTCCCTAAGGGTTGGGGATGACCCTTGGGTTTGTCTTTGGGTGTTACCTTTAGTGTCTCTCTGTGTCCCTATCTGTTACAGTCTCCTAAAGTATCCTCATAAAGTCACCTCCTAACGTCCATCCTAAAGCCAACACCTAAAGCCTACACCTAAAGACCCATCAAGTCAACGCCTATCTTAAAGTTTAAACATAAAGACCAGACCTAAAGACCAGACCTAA                                                                                                                                                                                                                                                                                    >  1:194555/1‑301`  
                      `GTACGGACCTAAAGTT‑CCCCAATA‑GGGGGTACCTAAAGCCCAGCCAATCACCTAAAGTCAACCTTCGGTTGACCTTGAGGGTTCCCTAAGGGTTGGGGATGACCCTTGGGTTTGTCTTTGGGTGTTACCTTGAGTGTCTCTCTGTGTCCCTATCTGTTACAGTCTCCTAAAGTATCCTCCTAAAGTCACCTCCTAACGTCCATCCTAAAGCCAACACCTAAAGCCTACACCTAAAGACCCATCAAGTCAACGCCTATCTTAAAGTTTAAACATAAAGACAAGACCTAAAGACCAGACCTAA                                                                                                                                                                                                                                                                                    >  1:2006/1‑301`  
                        `aaGGACCTAAAGTT‑CACCCATA‑GGGGGTACCTAAAGCCCAGCCAATCACCTAAAGTCAACCTTCGGTTGCCCTTGAGGGTTCCCTAAGGGTTGGGGATGACCCTTGGGTTTGTCTTTGGGTGTTACCTTGAGTGTCTCTCTGTGTCCCTATCTGTTACAGTCTCCTAAAGTATCCTCCTAAAGTCACCTCCTAACGTCCATCCTAAAGCCAACACCTAAAGCCTACACCTAAAGACCCATCAAGTCAACGCCTATCTTAAAGTTTAAACATAAAGACCAGACCTAAAGACCAGACCTAAAG                                                                                                                                                                                                                                                                                  <  1:212368/299‑1`  
                        `aaGGACCTAAAGTG‑CCCCCATA‑GGGGGTACCTAAAGCCCAGCCAATCACCTAAAGTCAACCTTCGTTTGACCTTGAGGGTTCCCTAAGGGTTGGGGATGACCCTTGGGTTTGTCTTTGGGTGTTGCCTTGAGTGTCTCTCTGTGTCCCTATCTGTTACAGTCTCCTAAAGTATCCTCCTAAAGTCACCTCCTAACGTCCATCCTAAAGCCAACACCTAAAGCCTACACCTAAAGACCCATCAAGTCAACGCCTATCTTAAAGTTTAAACATAAAGTCCAGACCTAAAGACCAGACCTAAAG                                                                                                                                                                                                                                                                                  <  1:564446/299‑1`  
                        `ACGTACCTAAAGTT‑CCCCCATA‑GGGGGTACCTAAAGCCCAGCCACTCACCTAAAGTCAACCTTCGGTTGACCTTGAGGGTTCCCTAAGGGTTGGGGATGACCCTTGGGTTTGTCTTTGGGTGTTACCTTGAGTGTCTCTCTGTGTCCCTATCTGTTACAGTCTCCTAAAGTATCCTCCTAAAGTCACCTCCTAACGTCCATCCTAAAGCCAACACCTAAAGCCTACACCTAAAGACCCATCAAGTCAACGCCTATCTTAAAGTTTAAACATAAAGACCAGACCTAAAGACCAGACCTAAAG                                                                                                                                                                                                                                                                                  <  1:348990/301‑1`  
                        `ACGGACCTAAAGTT‑CCCCCATA‑GGGGGTACCTAAAGCCCAGCCAATCACCTAAAGTCAACCTTCGGTTGACCTTGAGTGTTCCCTAAGGGTTGGGGATGACCCTTGGGTTTGTCTTTGGGTGTTACCTTGAGTGTCTCTCTGTGTCCCTATCTGTTACAGTCTCCTAAAGTATCCTCCTAAAGTCACCTCCTAACGTCCATCCTAAAGCCAACACCTAAAGCCTACACCTAAAGACCCATCAAGTCAACGCCTATCTTAAAGTTTAAACATAAAGACCAGACCTAAAGACCAGACCTAAAG                                                                                                                                                                                                                                                                                  <  1:305429/301‑1`  
                        `ACGGACCTAAAGTT‑CCCCCATA‑GGGGGTACCTAAAGCCCAGCCAATCACCTAAAGTCAACCTTCGGTTGACCTTGAGGGTTCCCTAAGGGTTGGGGATGACCCTTGGGTTTGTCTTTGGGTGTTACCTTGAGTGTCTCTCTGTGTCCCTATCTGTTACAGTCTCCTAAAGTATCCTCCTAAAGTCACCTCCTAACGTCCATCCTAAAGCCAACACCTAAAGCCTACACCTAAAGACCCATCAAGTCAACGCCTATCTTAAAGTTTAAACATAAAGACCAGACCTAAAGACCAGACCTAAAG                                                                                                                                                                                                                                                                                  <  1:30156/301‑1`  
                        `ACGGACCTAAAGTT‑CCCCCATA‑GGGGGTACCTAAAGCCCAGCCAATCACCTAAAGTCAACCTTCGGTTGACCTTGAGGGTTCCCTAAGGGTTGGGGATGACACTTGGGTTTGTCTTTGGGTGTTACCTTGAGTGTCTCTCTGTGTCCCTATCTGTTACAGTCTCCTAAAGTATCCTCCTAAAGTCACCTCCTAACGTCCATCCTAAAGCCAACACCTAAAGCCTACACCTAAAGACCCATCAAGTCAACGCCTATCTTAAAGTTTAAACATAAAGACCAGACCTAAAGACCAGACCTAAAG                                                                                                                                                                                                                                                                                  <  1:344307/301‑1`  
                         `tgtgtataagagacagCCCATA‑GGGGGTACCTAAAGCCCAGCCAATCACCTAAAGTCAACCTTCGGTTGACCTTGAGGGTTCCCTAAGGGTTGGGGATGACCCTTGGGTTTGTCTTTGGGTGTTAACTTGAGTGTCTCTCTGTGTCCCTATCTGTTACAGTCTCCTAAAGTATCCTCCTAAAGTCACCTCCTAACGTCCATCCTAAAGCCAACACCTAAAGCCTACACCTAAAGACCCATCAAGTCAACGCCTATCTTAAAGTTTAAACATAAAGACCAGACCTAAAGACCAGACCTAAAG                                                                                                                                                                                                                                                                                  <  1:485481/285‑1`  
                         `tgtataagagacagCCCCCATA‑GGGGGTACCTAAAGCCCAGCCAATCACCTAAAGTCAACCTTCGGTTGACCTTGAGGGTTCCCTAAGGGTTGGGGATGACCCTTGGGTTTGTCTTTGGGTGTTACCTTGAGTGTCTCTCTGTGTCCCTATCTGTTACAGTCTCCTAAAGTATCCTCCTAAAGTCACCTCCTAACGTCCATCCTAAAGCCAACACCTAAAGCCTACACCTAAAGACCCATCAAGTCAACGCCTATCTTAAAGTTTAAACATAAAGACCAGACCTAAAGACCAGACCTAAAG                                                                                                                                                                                                                                                                                  <  1:607896/287‑1`  
                         `tgtataagagacagCCCCCATA‑GGGGGTACCTAAAGCCCAGCCAATCACCTAAAGTCAACCTTCGGTTGACCTGGAGGGTTCCCTAAGGGTTGGGGATGACCCTTGGGTTTGTCTTTGGGTGTTACCTTGAGTGTCTCTCTGTGTCCCTATCTGTTACAGTCTCCTAAAGTATCCTCCTAAAGTCACCTCCTAACGTCCATCCTAAAGCCAACACCTAAAGCCTACACCTAAAGACCCATCAAGTCAACGCCTATCTTAAAGTTTAAACATAAAGACCAGACCTAAAGACCAGACCTAAAG                                                                                                                                                                                                                                                                                  <  1:622400/287‑1`  
                          `ggcCCTAGAGTT‑CCCCCATA‑GGGGGTACCTAAAGCCCAGCCATTCCCCTAAAGTCAACCTTCGGTTGACCTTGAGGGTTCCCTAAGGGTTGGTGATGACCCTTGGGTTTGTCTTTGGGTTTTACCTTGAGTGTCTCTCTGTGTCCCTATCTGTTACAGTCTCCTAAAGTATCCTCCTAAAGTCACCTCCTAACGTCCATCCTAAAGCCAACACCTAAAGCCTACACCTAAAGACCCATCAAGTCAACGCCTATCTTAAAGTTTAAACATAAAGACCAGACCTAAAGACCAGACCTAAAGAC                                                                                                                                                                                                                                                                                <  1:428979/298‑1`  
                          `GGACCTAAAGTT‑CCCCCATA‑GGGGGTACCTAAAGCCCAGCCAATCACCTAAAGTCAACCTTCGGTTGACCTTGAGGGTTCCCTAAGGGTTGGGGATGACCCTTGGGTTTGTCTTTGGGTGTTACCTTGAGTGTCTCTCTGTGTCCCTATCTGTTACAGTCTCCTAAAGTATCCTCCTAAAGTCACCTCCTAACGTCCATCCTAAAGCCAACACCTAAAGCCTACACCTAAAGACCCATCAAGTCAACGCCTATCTTAAAGTTTAAACATAAAGACCAGACCTAAAGACCAGACCTAAAGAC                                                                                                                                                                                                                                                                                <  1:392068/301‑1`  
                          `GGACCTAAAGTT‑CCCCCATA‑GGGGGTACCTAAAGCCCAGCCAATCACCTAAAGTCAACCTTCGGTTGACCTTGAGGGTTCCCTAAGGGTTGGGGATGACCCTTGGGTTTGTCTTTGGGTGTTACCTTGAGTGTCTCTCTGTGTCCCTAGCTGTTACAGTCTCCTAAAGTATCCTCCTAAAGTCACCTCCTAACGTCCATCCTAAAGCCAACACCTAAAGCCTACACCTAAAGACCCATCAAGTCAACGCCTATCTTAAAGTTTAAACATAAAGACCAGACCTAAAGACCAGACCTAAAGAC                                                                                                                                                                                                                                                                                <  1:519319/301‑1`  
                          `GGACCTAAAGGT‑CCCCCATA‑GGGGGTACCTAAAGCCCAGCCAATCACCTAAATTCAACCTTCGGTTGACCTTGAGGGTTCCCTAAGGGTTGGGGATGACCCTTTGGTTTGTCTTTGGGTGTTACCTTGAGTGTCTCTCTGTGTCCCTATCTGTTACAGTCTCCTAAAGTATCCTCCTAAAGTCACCTCCTAACGTCCATCCTAAAGCCAACACCTAAAGCCTACACCTAAAGACCCATCAAGTCAACGCCTATCTTAAAGTTTAAACATAAAGACCAGACCTAAAGACCAGACCTAAAGAC                                                                                                                                                                                                                                                                                <  1:377438/301‑1`  
                          `GGACCGAAACTT‑CCCCCATA‑GGGGGTACCTAAAGCCCAGCCAATCACCTAAAGTCAACCTTCTGTTTACCTTGAGGTTTCCCTAAGGGTTGGGTATGACCCTTTGGTTTGTCTTTGGGTGTTACCTTGAGTTTCTCTCTGTGTCCCTATCTGTTACAGTCTCCTAAAGTATCCTCCTAAAGTCACCTCCTAACGTCCATCCTAAAGCCAACACCTAAAGCCTACACCTAAAGACCCATCAAGTCAACGACTATCTTAAAGTTTAAACATAAAGACCAGACCTAAAGACCAGACCTAAAGAC                                                                                                                                                                                                                                                                                <  1:35983/301‑1`  
                           `GACCTAAAGTT‑CCCCCATAGGGGGGTACCTAAAGCCCAGCCAATCACCTAAAGTCAACCTTCGGTTGACCTTGAGGGTTCCCTAAGGGTTGGGGATGACCCTTGGGTTTGTCTTTGGGTGTTACCTTGAGTGTCTCTCTGTGTCCCTATCTGTTACAGTCTCCTAAAGTATCCTCCTAAAGTCACCTCCTAACGTCCATCCTAAAGCCAACACCTAAAGCCTACACCTAAAGACCCATCAAGTCAACGCCTATCTTAAAGTTTAAACATAAAGACCAGACCTAAAGACCAGACCTAACGAa                                                                                                                                                                                                                                                                                >  1:107827/1‑300`  
                           `GACCTAAAGTT‑CCCCCATA‑GGGGGTACCTAAAGCCCAGCCAATCACCTAAATTCAACCTTCGGTTGACCTTGAGGGTTCCCTACGGGTTGGGGATGACCCTTGGGTTTGTCTTTGGCTGTTACCTTGAGTGCCGCTCTGTGTCCCTATCTGTTACAGTCTCCTACAGTCTCCTCCTAAAGTCACCTCCTAACGTCCATCCTAAAGCCAACCCCTAAAGCCTACACCTAAAGACCCATCTAGTCCACGCCTATCTTAAAGATTAACCATAAAGACCAAACCTAAACACCAAACCTGAActct                                                                                                                                                                                                                                                                               >  1:653287/1‑297`  
                           `GACCTAAAGTT‑CCCCCATA‑GGGGGTACCTAAAGCCCAGCCAATCACCTAAAGTCAACCTTCGGTTGACCTTGAGGGTTCCCTAAGGGTTGGGGATGACCCTTGGGTTTGTCTTTGGGTGTTACCTTGAGTGTCTCTCTGTGTCCCTATCTGTTACAGTCTCCTAAAGTATCCTCCTAAAGTCACCTCCTAACGTCCATCCTAAAGCCAACACCTAAAGCCTACACCTAAAGACCCATCAAGTCAACGCCTATCTTAAAGTTTAAACATAAAGACCtgtctcttatacacatctccgagccc                                                                                                                                                                                                                                                                               >  1:225603/1‑275`  
                           `GACCTAAAGTT‑CCCCCATA‑GGGGGTACCTAAAGCCCAGCCAATCACCTAAAGTCAACCTTCGGTTGACCTTGAGGGTTCCCTAAGGGTTGGGGATGACCCTTGGGTTTGTCTTTGGGTGTTACCTTGAGTGTCTCTCTGTGTCCCTATCTGTTACAGTCTCCTAAAGTATCCTCCTAAAGTCACCTCCTAACGTCCATCCTAAAGCCAACACCTAAAGCCTACACCTAAAGACCCATCAAGTCAACGCCTATCTTAAAGTTTAAACATAAAGACCAGACCTAAAGctgtctcttatacacc                                                                                                                                                                                                                                                                               >  1:468631/1‑285`  
                           `GACCTAAAGTT‑CCCCCATA‑GGGGGTACCTAAAGCCCAGCCAATCACCTAAAGTCAACCTTCGGTTGACCTTGAGGGTTCCCTAAGGGTTGGGGATGACCCTTGGGTTTGTCTTTGGGTGTTACCTTGAGTGTCTCTCTGTGTCCCTATCTGTTACAGTCTCCTAAAGTATCCTCCTAAAGTCACCTCCTAACGTCCATCCTAAAGCCAACACCTAAAGCCTACACCTAAAGACCCATCAAGTCAACGCCTATCTTAAAGTTTAAACATAAAGACCAGACCTAAAGACCAGACCTgtctctt                                                                                                                                                                                                                                                                               >  1:298891/1‑294`  
                           `GACCTAAAGTT‑CCCCCATA‑GGGGGTACCTAAAGCCCAGCCAATCACCTAAAGTCAACCTTCGGTTGACCTTGAGGGTTCCCTAAGGGTTGGGGATGACCCTTGGGTTTGTCTTTGGGTGTTACCTTGAGTGTCTCTCTGTGTCCCTATCTGTTACAGTCTCCTAAAGTATCCTCCTAAAGTCACCTCCTAACGTCCATCCTAAAGCCAACACCTAAAGCCTACACCTAAAGACCCATCAAGTCAACGCCTATCTTAAAGTTTAAACATAAAGACCAGACCTAAAGACCAGACCTAAcgctg                                                                                                                                                                                                                                                                               >  1:502333/1‑296`  
                           `GACCTAAAGTT‑CCCCCATA‑GGGGGTACCTAAAGCCCAGCCAATCACCTAAAGTCAACCTTCGGTTGACCTTGAGGGTTCCCTAAGGGTTGGGGATGACCCTTGGGTTTGTCTTTGGGTGTTACCTTGAGTGTCTCTCTGTGTCCCTATCTGTTACAGTCTCCTAAAGTATCCTCCTAAAGTCACCTCCTAACGTACATCCTAAAGCCAACACCTAAAGCCTACACCTAAAGACACATCAAGTCAACGCCTATCTTAAAGTTTAAACATAAAGACCctgtctcttatacacatctccgagcc                                                                                                                                                                                                                                                                               >  1:419820/1‑275`  
                           `GACCTAAAGTT‑CCCCCATA‑GGGGGTACCTAAAGCCCAGCCAATCACCTAAACTCAACCTTCGGTTTACCTTGAGGGTTCCCTAAGGGTTGGGGATGTCCCTTGGGTTTGTCTTTGGGTGTTACCTTGAGTGTCTCTCTGTGTCCCTATCTGCTACAGTCTCCTAAAGTATCCTCCTAAATTCACCTCCTAACGTCCATCCTAAAGCGAACACCTAAAGCCTACACCTAAAGACCCCTCAAGTCAACGCCTATCTTAAAGTTTAAACATAAAGACCAGACCTAAAGACCAGAACTACAGctg                                                                                                                                                                                                                                                                               >  1:258715/1‑298`  
                                `AAAGTT‑CCCCCATA‑GGGGGTACCTAAAGCCCAGCCAATCACCTAAAGTCAACCTTCGGTTGACCTTGAGGGTTCCCTAAGGGTTGGGGATGACCCTTGGGTTTGTCTTTGGGTGTTACCTTGAGTGTCTCTCTGTGTCCCTATCTGTTACAGTCTCCTAAAGTATCCTCCTAAAGTCACCTCCTAACGTCCATCCTAAAGCCAACACCTAAAGCCTACACCTAAAGACCCATCAAGTCAACGCCTATCTTAAAGTTTAAACATAAAGACCAGACCTAAAGACCctgtctcttatacacatc                                                                                                                                                                                                                                                                          >  1:120835/1‑283`  
                                 `AAGTT‑CCCCCATA‑GGGGGTACCTAAAGCCCAGCCAATCACCTAAAGTCAACCTTCGGTTGACCTTGAGGGTTCCCTAAGGGTTGGGGATGACCCTTGGGTTTGTCTTTGGGTGTTACCTTGAGTGTCTCTCTGTGTCCCTATCTGTTACAGTCTCCTAAAGTATCCTCCTAAAGTCACCTCCTAACGTCCATCCTAAAGCCAACACCTAAAGCCTACACCTAAAGACCCATCAAGTCAACGCCTATCTTAAAGTTTAAACATAAAGACCAGACCTAAAGACCAGACCTAAAGctgtctctt                                                                                                                                                                                                                                                                         >  1:339561/1‑292`  
                                   `GTT‑CCCCCATA‑GGGGGTACCTAAAGCCCAGCCAATCACCTAAAGTCAACCTTCGGTTGACCTTGAGGGTTCCCTAAGGGTTGGGGATGACCCTTGGGTTTGTCTTTGGGTGTTACCTTGAGTGTCTCTCTGTGTCCCTATCTGTTACAGTCTCCTAAAGTATCCTCCTAAAGTCACCTCCTAACGTCCATCCTACAGCCAACACCTAAAGCCTACACCTAAAGACCCATCAAGTCAACGCCTATCTTACAGTTTAAACATAAAGACCAGACCTAAAGACCAGACCTAAAGctgtctcttat                                                                                                                                                                                                                                                                       >  1:451998/1‑290`  
                                   `GTT‑CCCCCATA‑GGGGGTACCTAAAGCCCAGCCAATCACCTAAAGTCAACCTTCGGTGGACCTTGAGGGTTCCCTAAGGGTTGGGGATGATCCTTGGGTTTGTCATTGGGTGTTACCTTGAGTGTCTCTCTGTGTCCCTATCTGTTACAGTCTCCTAAAGTATCCTCCTAAAGTCACCTCCTAACGTCCATCCTAAAGCCAACAACTAAAGCCTACACCTAAAGACCCATCAAGTCAACGCCTATCTTAAAGTTTAAACATAAAGACCACAACTAAAAAACAAACCTAAAAACCtgtatctt                                                                                                                                                                                                                                                                       >  1:313207/1‑293`  
                                          `CCATA‑GGGGGTACCTAAAGCCCAGCCAATCACCTAAAGTCAACCTTCGGTTGACCTTGAGGGTTCCCTAAGGGTTGGGGATGACCCTTGGGTTTGTCTTTGGGTGTTACCTTGAGTGTCTCTCTGTGTCCCTATCTGTTACAGTCTCCTAAATTATCCTCCTAAAGTCACCTCCTAACGTCCATCCTAAAGCCAACACCTAAAGCCAACACCTAAAGACCCATCAAATAAACTCCTATCTTAAAGTTTAAACATAAAGACCACACCTAAAGACCACACCTAAAGctgtactttataccaat                                                                                                                                                                                                                                                                 >  1:511284/1‑284`  
                                            `ATA‑GGGGGTACCTAAAGCCCAGCCAATCACCTAAAGTCAACCTTCGGTTGACCTTGAGGGTTCCCTAAGGGTTGGGGATGACCCTTGGGTTTGTCTTTGGGTGTTACCTTGAGTGTCTCTCTGTGTCCCTATCTGTTACAGTCTCCTAAAGTATCCTCCTAAAGTCACCTACTAACGTCCATCCTAAAGCCAACACCTAAAGCCTACACCTAAAGACCCATCAAGTCAACGCCTATCTTAAAGTTTAAACATAAAGACCAGACCTAAAGACCAGACCTAAAGACCtgtctcttatacacat                                                                                                                                                                                                                                                               >  1:137357/1‑285`  

                                                                                                                                                                                                                                                                                                        `|||||||||||||`                                                                                                                                                                                                                                                                                                    
`‑‑‑‑‑‑‑‑‑‑‑‑‑TCTCACAGTGTACGGACCTAAAGTT‑CCCCCATA‑GGGGGTACCTAAAGCCCAGCCAATCACCTAAAGTCAACCTTCGGTTGACCTTGAGGGTTCCCTAAGGGTTGGGGATGACCCTTGGGTTTGTCTTTGGGTGTTACCTTGAGTGTCTCTCTGTGTCCCTATCTGTTACAGTCTCCTAAAGTATCCTCCTAAAGTCACCTCCTAACGTCCATCCTAAAGCCAACACCTAAAGCCTACACCTAAAGACCCATCAAGTCAACGCCTATCTTAAAGTTTAAACA‑‑‑‑‑‑‑‑‑‑‑‑‑‑‑‑‑‑‑‑‑‑‑‑‑‑‑‑‑‑‑‑‑‑‑‑‑‑‑‑‑‑‑‑‑‑‑‑‑‑‑‑‑‑‑‑‑‑‑‑‑‑‑‑‑‑‑‑‑‑‑‑‑‑‑‑‑‑‑‑‑‑‑‑‑‑‑‑‑‑‑‑‑‑‑‑‑‑‑‑‑‑‑‑‑‑‑‑‑‑‑‑‑‑‑‑‑‑‑‑‑‑‑‑‑‑‑‑‑‑‑‑‑‑‑‑‑‑‑‑‑‑‑‑‑‑‑‑‑‑‑‑‑‑‑‑‑‑‑‑‑‑‑‑‑‑‑‑‑‑‑‑‑‑‑‑‑‑‑‑‑‑‑‑‑‑‑‑‑‑‑‑‑‑‑‑‑‑‑‑‑‑‑‑‑‑‑‑‑‑‑‑‑‑‑‑‑‑‑‑‑‑‑‑‑‑‑‑‑‑‑‑‑‑‑‑‑‑‑‑‑‑‑‑‑‑‑‑‑‑‑‑‑‑‑‑‑‑‑‑‑‑‑‑‑‑‑‑‑‑‑‑‑‑‑‑‑‑‑‑‑‑‑‑‑‑‑‑‑‑‑‑‑‑‑‑‑‑‑‑‑‑‑  >  NC_001604.1/1‑281`  
`‑‑‑‑‑‑‑‑‑‑‑‑‑‑‑‑‑‑‑‑‑‑‑‑‑‑‑‑‑‑‑‑‑‑‑‑‑‑‑‑‑‑‑‑‑‑‑‑‑‑‑‑‑‑‑‑‑‑‑‑‑‑‑‑‑‑‑‑‑‑‑‑‑‑‑‑‑‑‑‑‑‑‑‑‑‑‑‑‑‑‑‑‑‑‑‑‑‑‑‑‑‑‑‑‑‑‑‑‑‑‑‑‑‑‑‑‑‑‑‑‑‑‑‑‑‑‑‑‑‑‑‑‑‑‑‑‑‑‑‑‑‑‑‑‑‑‑‑‑‑‑‑‑‑‑‑‑‑‑‑‑‑‑‑‑‑‑‑‑‑‑‑‑‑‑‑‑‑‑‑‑‑‑‑‑‑‑‑‑‑‑‑‑‑‑‑‑‑‑‑‑‑‑‑‑‑‑‑‑‑‑‑‑‑‑‑‑‑‑‑‑‑‑‑‑‑‑‑‑‑‑‑‑‑‑‑‑‑‑‑‑‑‑‑‑‑‑‑‑‑‑‑‑‑‑‑‑‑‑‑‑‑‑‑‑‑‑‑‑‑‑‑‑‑‑‑‑‑‑‑‑‑‑‑‑‑‑‑‑‑‑‑‑‑‑‑‑‑‑‑‑‑‑‑‑‑‑‑‑TAAAGACCAGACCTAAAGACCAGACCTAAAGACACTACATAAAGACCAGACCTAAAGACGCCTTGTTGTTAGCCATAAAGTGATAACCTTTAATCATTGTCTTTATTAATACAACTCACTATAAGGAGAGACAACTTAAAGAGACTTAAAAGATTAATTTAAAATTTATCAAAAAGAGTATTGACTTAAAGTCTAACCTATAGGATACTTACAGCCATCGAGAGGGACACGGCGAATAGCCATCCCAATCGACACCGGGGTCAACCGGATAAGTAGACAGCCTGATAAGT  >  NC_001604.1/282‑571`

  

Alignment Legend

Aligned base mismatch/match (shaded by quality score): `ATCG/ATCG` `< 3 ≤` `ATCG/ATCG` `< 10 ≤` `ATCG/ATCG` `< 21 ≤` `ATCG/ATCG` `< 34 ≤` `ATCG/ATCG` `< 38 ≤` `ATCG/ATCG`

Unaligned base: `atcg`    Masked matching base: `atcg`    Alignment gap: `‑`    Deleted base: `‑`

Reads not counted as support for junction

read\_name Not counted due to insufficient overlap past the breakpoint.

read\_name Not counted due to not crossing MOB target site duplication.
